# Convalescent human IgG, but not IgM, from COVID-19 survivors confers dose-dependent protection against SARS-CoV-2 replication and disease in hamsters

**DOI:** 10.3389/fimmu.2023.1138629

**Published:** 2023-03-21

**Authors:** Hannah A. D. King, Vincent Dussupt, Letzibeth Mendez-Rivera, Bonnie M. Slike, Ursula Tran, Nathan D. Jackson, Erica Barkei, Michelle Zemil, Emily Tourtellott-Fogt, Caitlin H. Kuklis, Sandrine Soman, Aslaa Ahmed, Maciel Porto, Christopher Kitajewski, Brittany Spence, Dalia Benetiene, Lindsay Wieczorek, Swagata Kar, Gregory Gromowski, Victoria R. Polonis, Shelly J. Krebs, Kayvon Modjarrad, Diane L. Bolton

**Affiliations:** ^1^ US Military HIV Research Program, Walter Reed Army Institute of Research (WRAIR), Silver Spring, MD, United States; ^2^ Emerging Infectious Diseases Branch, WRAIR, Silver Spring, MD, United States; ^3^ Henry M. Jackson Foundation for the Advancement of Military Medicine, Bethesda, MD, United States; ^4^ Veterinary Pathology Branch, WRAIR, Silver Spring, MD, United States; ^5^ Viral Diseases Branch, WRAIR, Silver Spring, MD, United States; ^6^ BIOQUAL, Inc., Rockville, MD, United States

**Keywords:** SARS-CoV-2, antibody, passive transfer, IgM, IgG, hamster

## Abstract

**Introduction:**

Antibody therapeutic strategies have served an important role during the COVID-19 pandemic, even as their effectiveness has waned with the emergence of escape variants. Here we sought to determine the concentration of convalescent immunoglobulin required to protect against disease from SARS-CoV-2 in a Syrian golden hamster model.

**Methods:**

Total IgG and IgM were isolated from plasma of SARS-CoV-2 convalescent donors. Dose titrations of IgG and IgM were infused into hamsters 1 day prior to challenge with SARS-CoV-2 Wuhan-1.

**Results:**

The IgM preparation was found to have ~25-fold greater neutralization potency than IgG. IgG infusion protected hamsters from disease in a dose-dependent manner, with detectable serum neutralizing titers correlating with protection. Despite a higher *in vitro* neutralizing potency, IgM failed to protect against disease when transferred into hamsters.

**Discussion:**

This study adds to the growing body of literature that demonstrates neutralizing IgG antibodies are important for protection from SARS-CoV-2 disease, and confirms that polyclonal IgG in sera can be an effective preventative strategy if the neutralizing titers are sufficiently high. In the context of new variants, against which existing vaccines or monoclonal antibodies have reduced efficacy, sera from individuals who have recovered from infection with the emerging variant may potentially remain an efficacious tool.

## 1 Introduction

The ongoing spread of severe acute respiratory syndrome coronavirus-2 (SARS-CoV-2) continues to devastate global health and the world economy. Immense efforts by the scientific community have led to the development and deployment of multiple therapeutic and prophylactic strategies, resulting in several effective vaccines, monoclonal antibodies and anti-virals (1, 2). However, the unabated evolution and emergence of SARS-CoV-2 variants of concern has raised concerns about the continued effectiveness of current COVID-19 mitigation strategies.

The development of more broadly effective vaccines and therapeutics will be aided by a thorough understanding of the immune correlates of protection for COVID-19. Current work suggests that neutralizing antibodies, detected in the peripheral blood, are a major contributor toward conferring protection from symptomatic infection and disease. Efficacy studies among vaccinated or convalescent individuals (3–5), or studies that evaluated passive transfer of convalescent plasma (6–8) into naïve individuals, have demonstrated that neutralizing antibody activity correlates with reduced disease. Other research has also suggested a role for Spike-specific binding antibodies (9–13), SARS-CoV-2 specific Th1 CD4+ T cells (14–17) or CD8+ T cell responses (17, 18) and type I interferons (19, 20).

Given the importance of neutralizing antibodies in the protection against COVID-19, a number of monoclonal antibodies have been advanced, authorized and approved as primary options for the treatment of SARS-CoV-2 infection (21). Such treatments have had particular utility in populations where vaccination rates remain low or in individuals with a poor response to vaccination (including immunocompromised individuals) experiencing breakthrough SARS-CoV-2 infection following vaccination. Monoclonal antibodies have been shown to reduce viral load in infected patients and reduce the incidence of poor clinical outcomes and mortality (22–24). Preclinical studies have also demonstrated a reduction in disease following passive transfer of polyclonal immunoglobulin preparations from vaccinated or infected individuals/animals (25–28). The passive transfer of polyclonal immunoglobulin from the plasma of convalescent individuals to hospitalized COVID-19 patients has achieved modest success in improving clinical outcomes (29–34). Efficacy improves when high neutralizing titer convalescent plasma is used (35, 36), further supporting neutralization as a correlate of protection from infection and disease from SARS-CoV-2.

While most studies investigating the activity of monoclonal or polyclonal preparations have focused on the IgG isotype, other isotypes may also contribute to protection. The multivalency of IgM allows for improved targeting of antigens with a low affinity interaction, prompting investigation of IgM for application against other pathogens with encouraging findings. For example, mucosal administration of an anti-HIV IgM mAb is able to protect against SHIV infection in monkeys (37). Clinical trials assessing therapeutic IgM for a variety of non-communicable diseases such as arthritis and cancer have shown good safety profiles, though efficacy has been limited to date, perhaps due to the use of low affinity, non-affinity matured IgM antibodies (38). IgM targeting SARS-CoV-2 has been reported to have greater neutralization potency than IgG, both for monoclonal antibodies (39) and polyclonal convalescent plasma (40). In a mouse model using a mouse-adapted SARS-CoV-2, passive transfer of a human monoclonal IgM specific for SARS-CoV-2 Spike glycoprotein achieved a greater reduction in viral loads than the matched IgG when administered therapeutically (41). Passive transfer of IgM also protected hamsters against helminth infection (42), but this model has not been used to our knowledge for SARS-CoV-2.

In this study we compared the efficacy of IgM and IgG polyclonal immunoglobulin preparations from plasma of human convalescent COVID-19 cases against SARS-CoV-2 (Wuhan-1) infection and associated pathology in Syrian golden hamsters, a model with a severe disease phenotype that does not require genetic modification of the host or virus to promote viral replication and disease (43, 44). In *in vitro* assays, IgM pooled from convalescent COVID-19 individuals displayed enhanced neutralization potency relative to IgG isolated from the same individuals. Passive transfer of IgG into hamsters 24 hours prior to SARS-CoV-2 challenge protected from disease in a dose-dependent manner. While similar efficacy was not observed following IgM administration, IgM transudation into the respiratory tract may have been limited relative to the IgG, likely preventing antiviral activity at the site of exposure.

## 2 Materials and methods

### 2.1 Human samples

Plasma from SARS-CoV-2 convalescent donors was obtained from a leukapheresis protocol (#1386H) approved by the Walter Reed Army Institute of Research institutional review board - and for which participants provided written informed consent or from StemExpress (Folsom, CA, USA). Samples were collected from males (n = 7) and females (n = 4) ranging in age from 31 to 71 years. Individuals donated plasma specimens approximately 3 to 8 weeks after laboratory-confirmed SARS-CoV-2 infection from March-May 2020, and had clinical presentations that ranged from asymptomatic-to-mild-to-moderate; none were hospitalized for their condition. Samples from four donors were selected for large scale purification based upon the titers of SARS-CoV-2 neutralizing antibodies and volume of plasma available, with plasma neutralization ID50 titers ranging from 761 to 5,850. All samples were deidentified prior to use. Pooled naïve human serum collected prior to the COVID-19 pandemic was obtained from Seracare (Milford, MA, USA). All authors have complied with the ethical regulations regarding these studies.

### 2.2 Purification of human polyclonal immunoglobulins from convalescent and normal donors

Small-scale pilot purification of IgG, IgM and IgA was performed on 1 ml of plasma using one-step affinity Protein G Sepharose (Cytiva), POROS CaptureSelect IgM and IgA (ThermoFisher Scientific) gravity-flow columns, respectively. For large scale purification, pooled convalescent plasma (600 ml from four donors) and normal human serum (1 L, Seracare#1830-0005) were heat-inactivated, centrifuged at 10,000×g for 2 h and filtered through a 0.8 µm cellulose nitrate membrane. Filtered material was further diluted 10:1 with 10X Phosphate-buffered saline (PBS) and loaded on either custom 500 ml bed volume Protein G Sepharose 4 Fast Flow (Cytiva) column or 200 ml bed volume POROS CaptureSelect IgM (ThermoFisher Scientific) column, for selective IgG and IgM affinity purification, respectively, and both run at low pressure settings on a NGC FPLC system (BioRad). For loading, material was allowed to re-circulate for at least 2-3 passages through the columns. Extensive washes were performed using 1X PBS pH 7.4 until UV_280nm_ reached baseline (2-3 column volumes). Elution was performed in reverse-flow mode using 0.5 M Acetic Acid, pH 3.0. Eluted material was quickly neutralized with 3 M Tris, concentrated by tangential flow filtration using a Vivaflow 200 cassette (Sartorius), buffer exchanged to 1X PBS pH 7.4 and sterile filtered. Quantitation of material was performed on a Nanodrop spectrophotometer using IgG setting or an extinction coefficient of 1.18 for IgM. Purity was assessed by SDS-PAGE and identity confirmed by western blot and ELISA analyses. Endotoxin levels were measured using a LAL assay (Lonza). Molecular weights of 150,000, 160,000 and 900,000 Da were used to calculate neutralization activities of the purified IgG, IgA and IgM, respectively.

### 2.3 Animal study design and procedures

Male and female Syrian golden hamsters were acquired from Envigo and housed at BIOQUAL, Inc. for the duration of the study. Animals were acclimatized for one week prior to study commencement. Three animal experiments were performed, termed Phase A, B, and C. In Phase A, 18 hamsters (8 weeks of age, 85-120 g, healthy and research naïve) were infused intraperitoneally with a single-dose of either 100 mg/kg convalescent IgG, 100 mg/kg naïve IgG or PBS; investigators were not blinded to the group allocations. A sample size of n=6/group was chosen to detect a predicted 1-log difference in viral loads between the groups, assuming a standard deviation of 0.5-log. All animals were included in the analysis. Animals were distributed into groups based on their weight and sex. 24 h post- infusion, animals were challenged intranasally with SARS-Related Coronavirus 2, isolate USA-WA1/2020 (BEI Resources NIAID, NIH, NR-53780, Lot# 70038893). Virus was stored at -80°C prior to use, thawed by hand and placed immediately on wet ice. Stock was diluted 1:10 in PBS prior to inoculation *via* the intranasal route (50 µl per nare, 1.99 x 10^4^ TCID_50_ total dose). To minimize potential confounders animals were challenged in the order in which they received the infusion. Serum samples and oral swabs were collected at baseline, and days 3, 6, 9, 12 and 14 following challenge. All animals were weighed daily as the primary outcome measure and monitored for clinical signs of disease. At study termination (day 14) animals were anesthetized with ketamine/xylazine, followed by euthanasia. Lungs and nares were collected for histopathology and virologic assays.

In Phase B, 36 hamsters (10 weeks of age, 90-135 g) were infused intraperitoneally with either 500, 250, 50 or 10 mg/kg convalescent IgG, 100 mg/kg naïve IgG or PBS (n=6/group). Study procedures followed those as outlined above in Phase A; however, animals were euthanized on day 7 post-challenge, and blood samples and oral swabs were additionally taken at this timepoint. In Phase C, 36 hamsters (8-11 weeks of age, 90-120 g) were infused intraperitoneally with either 100 or 50 mg/kg convalescent IgM or PBS (n=6/group). Study procedures followed those outlined above with euthanasia at day 7 post-challenge.

Research was conducted under an approved animal use protocol in an AAALAC accredited facility in compliance with the Animal Welfare Act and other federal statutes and regulations relating to animals and experiments involving animals and adheres to principles stated in the Guide for the Care and Use of Laboratory Animals, NRC Publication, 2011 edition. Animal protocols and procedures were reviewed and approved by the Animal Care and Use Committee of both the US Army Medical Research and Development Command (USAMRDC, proposal number DM170728) Animal Care and Use Review Office as well as the Institutional Animal Care and Use Committee of Bioqual, Inc. (protocol number 20-079). USAMRDC and Bioqual, Inc. are both accredited by the Association for Assessment and Accreditation of Laboratory Animal Care and are in compliance with the Animal Welfare Act and Public Health Service Policy on Humane Care and Use of Laboratory Animals.

### 2.4 Immunoglobulin measurements

#### 2.4.1 ELISA

Concentration and purity of immunoglobulin preparations, and post-infusion concentration of immunoglobulin in hamster serum samples were measured by total human IgG, IgM and IgA enzyme-linked immunosorbent assay (ELISA) according to manufacturer’s protocols (Invitrogen). Immunoglobulin from oral swabs was eluted and concentrated 3-4-fold using Amicon ultra centrifugal units. Data was collected on a VersaMax^®^ microplate reader using SoftmaxPro v.6 (Molecular Devices). Final quantitation was assessed against 4-parameter logistic standard curves using Prism v.9 (GraphPad). For ELISAs on material eluted from oral swabs, a pooled normal human saliva (Innovative Research) was used as a positive control.

#### 2.4.2 Multiplex antibody binding assay

A high-throughput bead-based antibody binding assay was performed as previously described (45, 46) with modifications to adapt to coronavirus antigens. Briefly, heat-inactivated plasma from convalescent donors or purified immunoglobulin preparations was diluted and loaded into 384-well assay plates by use of a Biomek NXP^®^ automated liquid handler (Beckman Coulter). A cocktail of 25 coronavirus antigens and 2 control proteins (HIV-1 antigens), obtained commercially (SinoBiological) or internally produced (see below), spanning spike S1 and S2 domains for all 7 human coronaviruses, were covalently coupled to uniquely coded magnetic microspheres (Luminex) per manufacturer’s protocol and added to the plate in a final volume of 50 μl/well. Following a 2 h incubation with vigorous shaking, microspheres were washed using a magnetic 384-well automated plate washer (Bio-Tek) to remove unbound sample. Microspheres were then resuspended with 0.5 μg/ml mouse anti-human IgG-PE (Southern Biotech), vortexed for 1 min with a microplate vortex at 3,000 rpm, sonicated for 1 min and then incubated with vigorous shaking for 1 h. After a final wash to remove unbound detection reagent, microspheres were resuspended in 40 μl sheath fluid (Luminex). Data was collected on a Bio-Plex^®^3D Suspension Array system (Bio-Rad) running xPONENT^®^ v.4.2 (Luminex). Signal to Noise (S/N) ratio were calculated by the dividing the MFI for each sample by either Ig-depleted healthy plasma or a negative control antibody (MZ4) according to the type of sample analyzed.

#### 2.4.3 SARS-CoV-2 pseudovirus neutralization assay

SARS-CoV-2 pseudovirions (pSV) were produced by co-transfection of HEK293T/17 cells with a pcDNA3.1 encoding SARS-CoV-2 S and an HIV-1 NL4-3 luciferase reporter plasmid (pNL4-3.Luc.R-E-, NIH AIDS Reagent Program). The S expression plasmid sequence was derived from the Wuhan Hu-1 strain (GenBank # NC_045512), which is also identical to the IL1/2020 and WA1/2020 strains. The S expression plasmid sequence was codon optimized and modified to remove the last 18 amino acids of the cytoplasmic tail to improve S incorporation into the pseudovirions and thereby enhance infectivity. Virions pseudotyped with the vesicular stomatitis virus (VSV) G protein were used as a negative control. Infectivity and neutralization titers were determined using ACE2-expressing HEK293 target cells (Integral Molecular) in a semi-automated assay format using robotic liquid handling (Biomek NXp Beckman Coulter). Convalescent human samples were diluted 1:40 in growth medium and serially diluted. Hamster serum samples were diluted 1:10 in growth medium and serially diluted. Then 25 μl/well was added to a white 96-well plate. Purified IgG and IgM were tested at a starting concentration of 1 mg/ml. An equal volume of diluted SARS-CoV-2 pSV was added to each well and plates were incubated for 1 h at 37°C. Target cells were added to each well (40,000 cells/well) and plates were incubated for an additional 48 hr. Luciferase activity was measured with the EnVision Multimode Plate Reader (Perkin Elmer) using the Bright-Glo Luciferase Assay System (Promega). Neutralization dose–response curves were fitted by nonlinear regression using the LabKey server, and the final titers are reported as the reciprocal of the dilution of plasma necessary to achieve 50% neutralization (ID50, 50% inhibitory dose or IC50, 50% inhibitory concentration) and 80% neutralization (ID80, 80% inhibitory dose or IC80, 80% inhibitory concentration). Assay equivalency was verified by participation in the SARS-CoV-2 Neutralizing Assay Concordance Survey (SNACS) run by the Virology Quality Assurance Program and External Quality Assurance Program Oversite Laboratory (EQAPOL) at the Duke Human Vaccine Institute, sponsored through programs supported by the National Institute of Allergy and Infectious Diseases, Division of AIDS.

### 2.5 Total and subgenomic messenger RNA quantification

Real-time quantitative PCR was carried out for total and subgenomic viral load RNA quantification from oral swabs, nares and lungs. Viral RNA was isolated from oral swabs using the Qiagen MinElute virus spin kit, and from tissues extracted using RNA-STAT 60 (Tel-test “B”), chloroform, precipitated and re-suspended in RNAse-free water. Positive controls for the amplification reaction were produced from RNA isolated from the applicable SARs-CoV-2 stock using the same procedure. Primers targeted the nucleocapsid (NC) gene of SARS-CoV-2 for measuring the total RNA, and to the envelope (E) gene for measuring subgenomic viral RNA (sgmRNA) (**Table S1**).

The PCR reaction contained 1x SensiFAST Probe No-ROX One-Step Mix buffer, reverse transcriptase and RiboSafe RNase Inhibitor, all from the TaqMan RT-PCR kit (Bioline), primers (600 nM) and probe (140 nM). Amplification was performed on an Applied Biosystems 7500 Sequence detector and amplified using the following program: 48°C for 30 min, 95°C for 10 min followed by 40 cycles of 95°C for 15 sec, and 1 min at 55°C. A standard curve was prepared with a range of 1 to 10^7^ copies/reaction for total RNA and 1 to 10^6^ copies/reaction for sgmRNA. The number of copies of RNA per ml was calculated by extrapolation from the standard curve, giving a practical range of 50 to 5 x 10^8^ RNA copies per gram tissue for total RNA and 50 to 5 x 10^7^ RNA copies per gram tissue for sgmRNA. All samples were tested in triplicate.

### 2.6 Histopathologic analysis

Necropsies were performed according to IACUC approved protocols at 7 or 14 days post-infection. Lungs were insufflated and perfused with 10% neutral-buffered formalin. Five tissue sections from each of the left lung lobes were used to evaluate the lung pathology. Sections were processed routinely into paraffin wax, then sectioned at 5 µm, and resulting slides were stained with hematoxylin and eosin. All tissue slides were evaluated by a board-certified veterinary anatomic pathologist blinded to study group allocations. Semi-quantitative scoring of pulmonary pathology was performed, with grading of hemorrhage, intra-alveolar edema, type II pneumocyte hyperplasia, mononuclear cellular infiltrates, polymorphonuclear cellular infiltrates, alveolar histiocytosis, thickened alveolar septa, alveolar necrosis, bronchioalveolar epithelial degeneration, bronchiolar epithelial hyperplasia, and interstitial collagenous deposition. Each finding was scored as follows: 0 - absent; 1 - minimal (<10% of tissue section affected); 2 - mild (11-25% of tissue section affected); 3 - moderate (26-50% of tissue section affected); 4 - marked (51-75% affected); 5- severe (>75% of tissue section affected).

### 2.7 Statistical analysis

Study outputs comprised of pathology scores and viral loads were compared across vaccination groups using the Kruskal-Wallis test in GraphPad Prism v9 software. Non-parametric pairwise comparisons between groups were made using the *post-hoc* Dunn’s test. Correlations between measurements were assessed using a nonparametric Spearman correlation. Statistical significance was preset at an alpha level of 0.05.

## 3 Results

### 3.1 Characterization and purification of IgG and IgM from convalescent individuals

Convalescent plasma was obtained from 11 individuals 17 to 59 days following a SARS-CoV-2 positive PCR test spanning March to May of 2020 (**Supplementary Figure S1A**). Most donors experienced mild to moderate symptoms; a few had asymptomatic infection. Plasma was tested for SARS-CoV-2 neutralization activity using a spike (S) pseudotyped lentivirus virion assay (PSV) against autologous SARS-CoV-2 (Wuhan-1). Consistent with previous reports (47, 48), we measured a wide range in neutralization activity from below the assay limit of detection to an ID50 titer > 5,000 in one donor. To evaluate the immunoglobulin isotype(s) responsible for the neutralization activity, we measured IgG, IgM and IgA binding activity to a panel of antigens, encompassing all domains of S, from SARS-CoV-2 and other human coronaviruses, using a bead-based multiplex assay. Binding antibody responses to SARS-CoV-2 antigens correlated with neutralization titer across all isotypes (**Supplementary Figure S1B**). The strongest correlation was observed with IgM binding responses (r=0.87), followed by IgG (r=0.84) and IgA (r=0.72). Binding of IgG to SARS-CoV (r=0.77) also associated with SARS-CoV-2 neutralization, consistent with high sequence homology between their respective S proteins and the cross-neutralization observed for some monoclonal antibodies (49, 50). As expected, weak or absence of correlation was observed between binding to the more distant MERS-CoV and other non-pathogenic human coronaviruses such as OC43, HKU1, NL63 and 229E and SARS-CoV-2 neutralization. To directly assess the neutralization activity of each isotype, we selected four donors based on their high neutralization titers and volume of plasma available (**Supplementary Figure S1C**) and selectively purified IgG, IgM and IgA from plasma. The IgM and IgG fractions were analyzed by SDS-PAGE electrophoresis under reducing and non-reducing conditions. In non-reducing conditions, purified IgM existed predominantly as a large band (>250 kDa), indicating the presence of higher order multimers, likely native pentamers (**Supplementary Figure S2A**). The purity of the immunoglobulin fractions, as assessed by ELISA, was 98.8% for IgM and 98.3% for IgG (**Table 1**; **Supplementary Figure S2B**). Neutralization activity was measured in the purified material. Purified IgM displayed the most potent neutralizing activity (10- to 60-fold higher than IgG), while IgA had the least potent neutralizing activity (**Supplementary Figure S1D**).

**Table 1:** Characteristics of purified IgG and IgM.

IgG purification							
Starting material	Yield (g/L)	IgG purity (%)	IgA (%)	IgM (%)	IgG (mg/mL)	Endotoxin (EU/mL)	SARS-CoV-2 neutralization (IC50 nM)
Naïve human serum pool	7.5	90.3	2.8	7.0	50.5	0.05	>1,000 (non-neutralizing)
COVID+ plasma pool	9.6	98.3	0.0	1.7	54.1	0.01	153.3
IgM purification							
Starting material	Yield (g/L)	IgM purity (%)	IgG (%)	IgA (%)	IgM (mg/mL)	Endotoxin (EU/mL)	SARS-CoV-2 neutralization (IC50 nM)
COVID+ plasma pool	0.55	98.8	0.0	1.1	11.5	0.41	5.5

### 3.2 Passive transfer of convalescent human IgG and IgM in hamsters

Given the strong neutralizing activity of both IgG and IgM, we evaluated the therapeutic potential of both antibody isotypes in a pathogenic SARS-CoV-2 animal model using human convalescent IgG and IgM. To obtain sufficient material for passive immunization, we pooled plasma from the four selected donors described above and sequentially purified IgG and IgM using isotype specific affinity purification. Control IgG was similarly purified from naïve healthy donor plasma sampled prior to 2019. Yields for SARS-CoV-2 convalescent IgG and IgM were 9.6 and 0.55 g/L, respectively (**Table 1**). The neutralization IC50 for purified convalescent polyclonal IgG and IgM preparations was 153 and 5.5 nM, respectively, with lower IC50 indicating increased neutralization potency of IgM compared to IgG.

72 Syrian golden hamsters (n=6 per group) were infused with a dose titration of the purified IgG or IgM from SARS-CoV-2 convalescent or naïve individuals, or with PBS in a series of three study phases (**Supplementary Figure S3**). Twenty-four hours following the infusion, animals were challenged with SARS-CoV-2 (WA1/2020; 1.99 x 10^4^ TCID_50_) *via* the intranasal route. Animals were followed for 14 (n=18, Phase A), or 7 (n=52, Phases B and C) days following challenge for immunologic, virologic and pathologic assessments.

### 3.3 SARS-CoV-2-specific antibody activity in hamster serum following passive transfer

**Figure 1:**
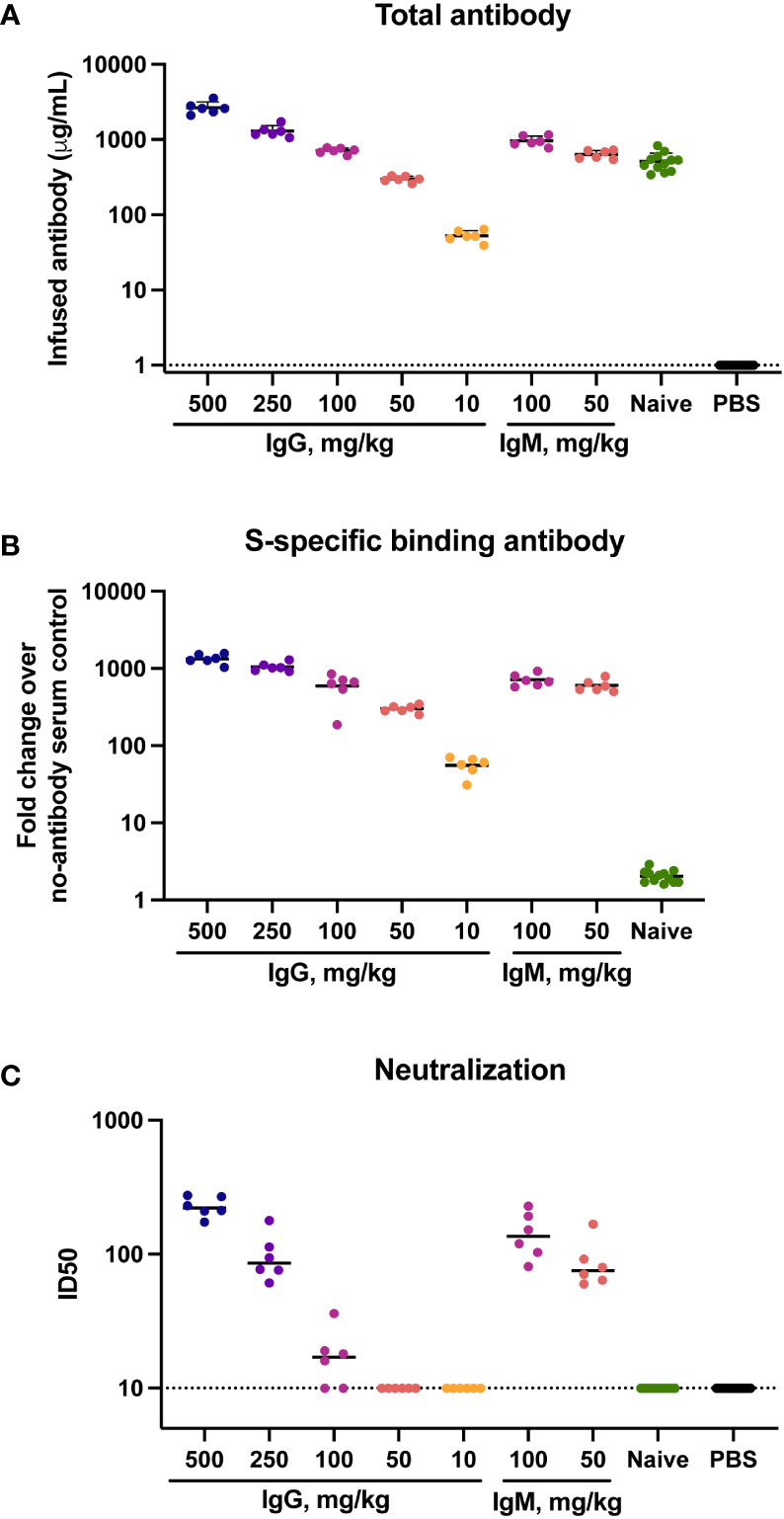
Total, binding and neutralizing antibody titers at time of challenge in serum. Titers of infused antibodies were measured in hamsters at the time of challenge (study day 0). **(A)** Serum total levels of the infused human antibodies as assessed by human IgG or IgM ELISA assays. **(B)** Serum SARS-CoV-2 specific IgG/IgM responses as assessed by multiplex antibody binding assay to the Hexapro stabilized spike protein **(C)** Serum SARS-CoV-2 S-specific pseudovirus neutralization. Virus neutralization reciprocal 50% inhibitory dilution (ID_50_) is shown. X-axis values indicate mg/kg of IgM or IgG administered to animals. Dotted lines indicate the lower limit of detection for the assay. Horizontal bars indicate group means.

To determine the concentration of infused immunoglobulin in animals following transfer total human immunoglobulin was measured in sera at the time of challenge by ELISA. Human IgG and IgM present in hamster sera was interpreted to reflect the amount of infused immunoglobulin. Geometric mean (GMT) IgG concentrations were 2,629 μg/ml in animals that received 500 mg/kg IgG, and 52 μg/ml in animals that received 10 mg/kg IgG (**Figure 1A**). Animals that received 100 mg/kg naïve IgG had a similar amount (499 μg/ml) of IgG to animals that received 100 mg/kg convalescent IgG (711 μg/ml). The animals that received IgM had a slightly higher amount of human immunoglobulin in sera than those that received a comparable amount of IgG, with infusion of 100 mg/kg IgM resulting in 955 μg/ml IgM at the time of challenge. No human immunoglobulin was detected in hamsters that received PBS, confirming specificity of the detection reagents for human IgG and IgM.

The presence of SARS-CoV-2-specific binding antibodies was measured by a multiplex antibody binding assay to the full S protein (**Figure 1B**) and to the S1 subunit and the receptor binding domain (RBD) and N-terminal domain (NTD) (**Supplementary Figure S4A-C**). SARS-CoV-2-specific antibody amount present at the time of challenge was consistent with the amount of total human immunoglobulin in serum, both exhibiting a clear dose response to the amount of infused immunoglobulin. Higher binding antibody titers were present in animals that received IgM compared to those that received IgG. Binding to the stabilized Hexapro full spike antigen (51) at a 50 mg/kg dose IgM had a 595-fold geometric mean increase over the PBS control animals, while 50 mg/kg IgG resulted in a 299-fold increase. No SARS-CoV-2 binding antibodies were detected in the animals that received naïve IgG.

Functional antibodies were assessed using a pseudovirus neutralization assay with the Wuhan-Hu-1 strain. Neutralization activity was present in sera of all animals that received 500 or 250 mg/kg IgG, with geometric mean ID50 titers of 226 and 94 respectively (**Figure 1C**). Four of six animals that received 100 mg/kg IgG had low but measurable neutralization titers, with a geometric mean ID50 of 21. Animals that received 100 mg/kg and 50 mg/kg IgM had ID50 GMTs of 137 and 83, respectively. These titers were higher than those observed in animals that received an equivalent dose of IgG, reflective of the higher neutralization potency of the parent IgM material.

To assess the presence of the infused material in a mucosal compartment, we measured human immunoglobulin in oral swab samples from a subset of hamsters. IgG was measured in a subset of four animals that received 500 or 250 mg/kg convalescent IgG. In all samples, human IgG concentrations exceeded the assay lower limit of detection (**Supplementary Figure S5**). A positive control of a pooled normal human ELISA sample had IgG levels exceeding those eluted from the hamster oral swabs. No measurable IgM was detected in the oral swabs of three animals that received 100 mg/kg IgM, suggesting a lack of human IgM transudation to this mucosal compartment. The limit of IgM detection was higher (15.6 ng/mL) than the IgG assay (1.6 ng/mL) and 2/4 IgG sample measurements fell below 15.6 ng/mL. Therefore, it is possible low levels of IgM were present and similar to those of IgG, but below the sensitivity of the IgM assay.

### 3.4 IgG infusion prior to challenge resulted in a dose-dependent protection from disease

**Figure 2:**
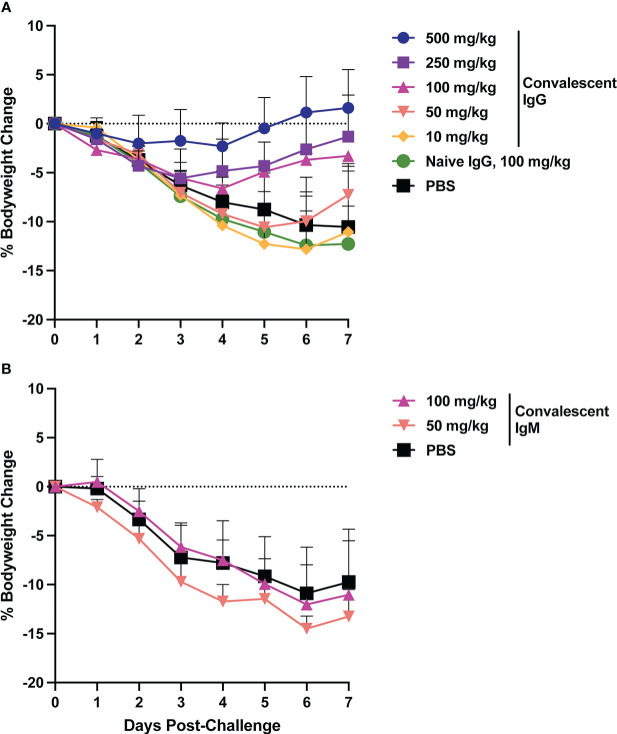
Body weight changes following SARS-CoV-2 challenge. Hamster weight was recorded daily in hamsters from the time of viral challenge until necropsy. Graphs show the mean body weight change from time of challenge for each group in the animals that received passively transferred IgG **(A)** or IgM **(B)**. Error bars indicate standard deviation.

**Figure 3:**
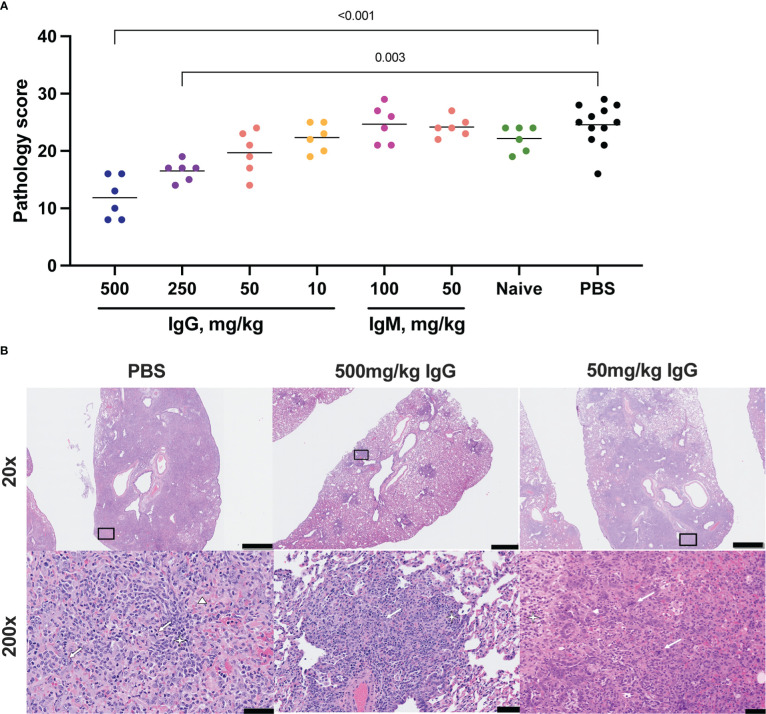
Histopathologic examination post-challenge. Lung tissues were collected at necropsy on day 7 post-challenge, fixed with neutral buffered formalin, and stained with hematoxylin and eosin (H&E) for standard microscopic examination. **(A)** H&E stained slides were scored for pathologic effects (see Methods) in SARS-CoV-2 challenged hamsters. Dots represent individual animals and the horizontal bar is the mean group score. Each group was compared to the control group that received no antibody infusion by Kruskal-Wallis test with Dunn’s posttest and bars indicate significant differences (P<0.05). **(B)** Representative lung tissue sections from the 7 day PBS control, 7 day 500 mg/kg convalescent IgG, and 7 day 50 mg/kg convalescent IgG, challenged hamsters in the columns as indicated. Rows are given by H&E at 20 and 200 times magnification power (20X and 200X, respectively). The black boxes in the top row indicate the area magnified in the bottom row. Interstitial pneumonia is characterized by inflammatory cellular infiltrates (star), type II pneumocyte hyperplasia (thick arrow), bronchiolar epithelial hyperplasia, and hemorrhage and edema (triangle). Scale bars: Top row, 1 mm; bottom row, 50 µm.

Animal bodyweight was measured daily following SARS-CoV-2 challenge to assess disease severity. In the control animals, peak weight loss of ~10% occurred on days 6-7 post-challenge (**Figure 2**). Infusion of SARS-CoV-2 convalescent IgG reduced animal weight loss, with greatest effects observed at the higher doses (**Figure 3A**). Transfer of 500 mg/kg IgG resulted in insignificant weight change (mean +1.1%) at day 6. Animals that received 250 or 100 mg/kg convalescent IgG were protected to a lesser extent (mean weight change of -2.6% and -3.7% at day 6 respectively), and animals that received 50 or 10 mg/kg IgG had weight change comparable to the control animals. Among animals followed out to 14 days post-challenge, weight loss peaked 6-7 days post-challenge among controls, followed by a gradual regaining of weight. All animals recovered their pre-challenge weights by day 14, with mean weight changes of 2.4, -0.1 and 0.9% in the 100 mg/kg convalescent IgG, naïve IgG and PBS groups respectively (**Supplementary Figure S6**).

Weight loss in control animals in the IgM phase of the experiment matched that of the control animals in the IgG phase, with a mean weight change of -10.9% at day 6. In contrast to IgG, infusion of convalescent IgM did not prevent weight loss, with mean weight changes of -12 and -14.5% at day 6 among 100 mg/kg and 50 mg/kg IgM recipient animals, respectively (**Figure 2B**).

### 3.5 IgG infusion associated with reduced lung pathology at necropsy

Lung pathology was assessed by routine hematoxylin and eosin (H&E) staining and semiquantitative scoring of histopathology 7 days post-SARS-CoV-2 challenge. The highest degree of pathology was present in the PBS and naïve IgG control animals (**Figure 3A**). All control animals developed evidence of moderate to marked interstitial pneumonia. The pneumonia was characterized by type II pneumocyte hyperplasia, alveolar hemorrhage and edema, alveolar inflammation and necrotic debris, thickening of alveolar septae, bronchiolar epithelial hyperplasia and increased numbers of pulmonary macrophages (including multinucleated giant cells) (**Figure 3B**). Pathology was most limited in animals that received 500 mg/kg convalescent IgG, although a significant improvement was also seen with 250 mg/kg IgG. There was no decrease in the mean pathology score among the convalescent IgM groups compared to either PBS or naïve IgG controls. The individual pathology findings that contribute to the overall pathology score replicated the pattern observed for the overall score (**Supplementary Figures S7A-C**), with dose-dependent reductions in type II pneumocyte hyperplasia, cellular infiltrates without alveolar histiocytosis and cellular infiltrates with alveolar histiocytosis following IgG infusion. Pathology in control animals 14 days post-challenge was more limited than that observed at day 7 (**Supplementary Figures S8A, B**). Nonetheless, 100 mg/kg convalescent IgG pre-treatment reduced pathology compared to the PBS animals (**Supplementary Figure S8A**).

### 3.6 IgG infusion was associated with lower SARS-CoV-RNA in the lower respiratory tract

**Figure 4:**
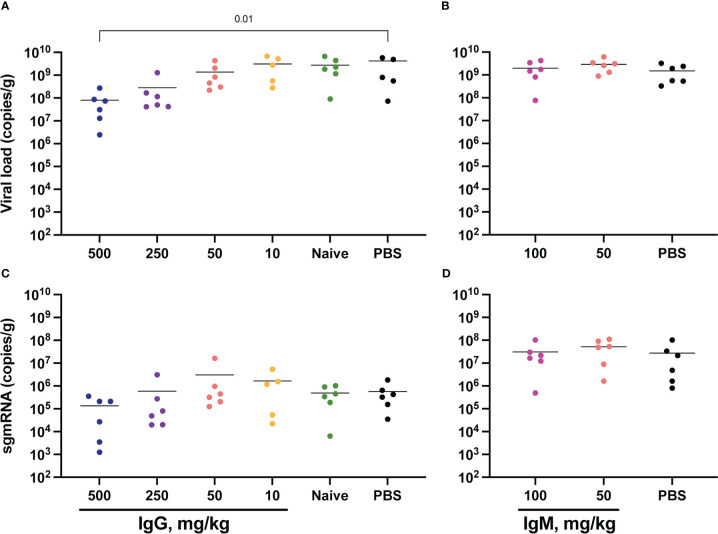
Lung viral loads post-challenge. Lung tissues were collected at necropsy on day 7 post-challenge for viral RNA extraction from one lung lobe. Total SARS-CoV-2 RNA was measured by PCR for IgG **(A)** and IgM **(B)** animals, and subgenomic RNA measured for IgG **(C)** and IgM **(D)** animals. Horizontal bars indicate the group means. Each group was compared to the control group that received no antibody infusion by Kruskal-Wallis test with Dunn’s posttest and bars indicate significant differences (P<0.05).

SARS-CoV-2 total RNA and subgenomic (sg) mRNA [considered a more specific indicator of viral replication (52, 53)] were measured following challenge to assess the viral burden in the respiratory tract. Seven days post-challenge, total SARS-CoV-2 RNA in the lower respiratory tract (as measured in the lungs) was diminished in animals that received 500 mg/kg IgG. There was also a trend towards reduced SARS-CoV-2 RNA in the animals that received 250 mg/kg IgG (**Figure 4A**). Other convalescent IgG dose groups did not differ from controls, nor did animals that received convalescent IgM (**Figure 4B**). Lung sgmRNA was similar in all groups (**Figures 4C, D**). sgmRNA was below the limit of detection in the lungs of most animals necropsied at day 14 (**Supplementary Figure S8C**).

SARS-CoV-2 RNA was also measured in the upper respiratory tract, specifically in the nares collected at day 7 post-challenge (**Supplementary Figure S9**). Here, both total RNA and sgmRNA were significantly lower in animals that received 50 mg/kg convalescent IgM (**Supplementary Figures S9B, D**). Nares SARS-CoV-2 RNA did not differ between controls and animals that received IgG measured at day 7 (**Supplementary Figures S9A, C**) and at day 14 only one animal had measurable sgmRNA in the nares (**Supplementary Figure S8D**). Oral swabs collected longitudinally following challenge also allowed for repeated measurement of SARS-CoV-2 RNA in the upper respiratory tract (**Supplementary Figure S10**). Similar to viral RNA in the nares, passive transfer of convalescent IgG did not impact either total or subgenomic RNA in this upper respiratory tract compartment.

### 3.7 Total infused immunoglobulin, SARS-CoV-2 binding and neutralizing titers correlate with protection from disease

**Figure 5:**
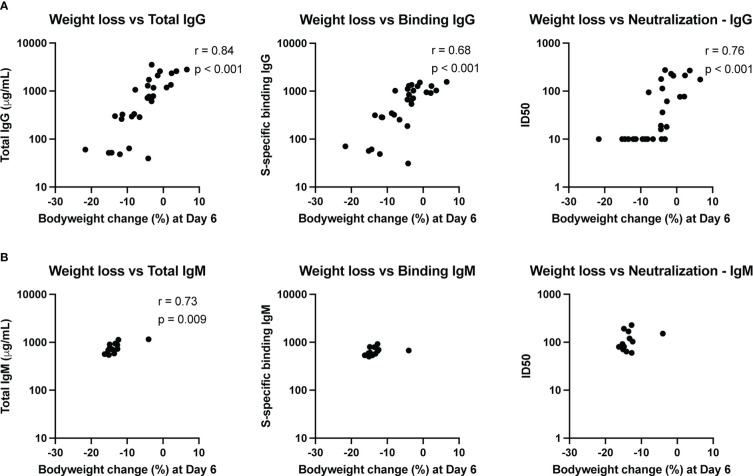
Immune correlates of protection. The relationship between total antibody levels, S-specific binding antibody and neutralizing antibodies at the time of challenge and weight change at day 6 post-challenge was assessed by a nonparametric Spearman correlation for animals that received convalescent IgG **(A)** or IgM **(B)**. This analysis did not include animals that received naïve IgG or no antibody infusion. Binding IgG values represent the fold-change over a no-antibody control.

To assess whether the amount of transferred convalescent immunoglobulin correlated with degree of protection against SARS-CoV-2 induced disease, we performed nonparametric Spearman correlations between quantitative serum antibody measurements at the time of challenge and maximum weight loss post-challenge (day 6) in animals infused with either IgG or IgM from convalescent individuals. Weight loss inversely correlated with total infused human IgG (non-antigen specific) concentration in hamster serum at the time of challenge, as well as with S-specific binding IgG and SARS-CoV-2 neutralizing antibody titers (**Figure 5A**). For neutralization activity, in general, ID50 values greater than 50 were protective from weight loss, and minimal weight loss was observed in all animals with neutralization activity above the assay LOD.

Since the animals that received convalescent IgM were not protected from bodyweight loss post-challenge, the variation in weight change for these animals was small. Nonetheless, total IgM was associated with less weight loss (**Figure 5B**), though the biological relevance of this correlation is unclear given the small dynamic range. Neither S-specific binding or neutralizing IgM titers correlated with weight loss.

## 4 Discussion

This study demonstrates that passively transferred purified IgG from SARS-CoV-2 convalescent donors is able to protect Syrian golden hamsters from SARS-CoV-2 disease in a dose-dependent manner. Viral replication in the upper respiratory tract was not suppressed by transfer of purified IgG at concentrations up to 500 mg/kg, with comparable virus present in the nares and oral cavity relative to controls after virus challenge. However, high doses of IgG protected animals from weight loss and lung pathology while reducing lower respiratory tract SARS-CoV-2 RNA compared to controls. Multiple pre-clinical vaccine studies in non-human primates and hamsters have observed similar effects on SARS-CoV-2 respiratory tract viral RNA levels whereby adaptive immune responses are more effective at limiting viral replication in the lower respiratory tract (54–57). Total human immunoglobulin, S-specific binding titers and neutralization activity in animal sera at the time of challenge all inversely correlated with weight loss. However, animals with measurable neutralization titers were most protected from disease. Other preclinical studies investigating passive transfer of polyclonal immunoglobulin preparations have shown similar results, with neutralization ID50 titers of approximately 50-400 required for protection from disease (25, 58, 59), underscoring the importance of maintaining strong neutralization against emerging variants. Work measuring neutralization titers in convalescent plasma has demonstrated that the presence of high neutralization activity in plasma correlates with the ability to neutralize VOCs, despite no previous exposure to the selected variants (60). In the setting of future emerging variants less sensitive to existing monoclonal antibodies treatment with sera from recent convalescent patients may serve as an effective prevention or therapeutic tool, particularly in the absence of other strategies.

This study also tested the protective efficacy of IgM isolated from the plasma of convalescent donors. Despite the higher *in vitro* neutralization activity of IgM than that of IgG, we did not observe the same *in vivo* protection mediated by IgM as with IgG. A significant reduction in SARS-CoV-2 RNA in the nares was observed in animals that received 50 mg/kg IgM, however this did not correspond to any reduction in disease. A similar observation was recently reported following administration of plasma depleted of IgG, which abrogated protection in K18-hACE2 transgenic mice, while IgA- plus IgM-depleted plasma did not (61). Efforts to quantify human immunoglobulin in oral swabs collected 24 hours post-infusion revealed the presence of human IgG but not IgM, suggesting that human IgM may not transudate as efficiently as IgG into mucosal sites of hamsters. IgM may be expected to traffic better to the mucosa than IgG, due to the presence of a joining (J) chain (62). While the J-chain is relatively conserved between humans and hamsters (38), the ability of human antibodies to traffic to mucosal sites in the hamster model has not been studied. Consequently, it is possible that IgM trafficking to the site of infection is impaired due to the species mismatch. However, the assay used for quantifying IgG had a ~10-fold lower limit of detection compared to the IgM assay. Therefore, IgM may have been present at mucosal sites albeit at very low levels that did not confer protection. It is possible that infusion of a higher dose of IgM may be efficacious. We did not assess the effectiveness of passive transfer of IgA in this study, as the IgA serum fraction exhibited lower neutralization activity than the IgM and IgG fractions. However, IgA mucosal transudation may be more efficient, augmenting its potential to mediate protection despite the decreased potency, and merits investigation in future work.

While a previous study demonstrated protection from SARS-CoV-2 following administration of a human IgM monoclonal antibody (41), this antibody was delivered intranasally, in contrast to the IP administration used here, therefore no trafficking of the IgM to the site of challenge was required. The translocation of IgM to the mucosa requires interaction between the polymeric immunoglobulin receptor (pIgR) and J-chain of the IgM and leads to the secretory component remaining associated with the IgM once in the mucosa (63). It is possible that even if the IgM effectively transudated to the mucosa, the attached species-mismatched secretory component may have inhibited the function of the human IgM in the mucosa. Human IgM receptors (FcµR) likely have low homology with hamster IgM receptors – amino acid sequence identity between human and mouse FcµR is 54% (64) – and this mismatch may limit activity. Additionally, the intranasal IgM delivery was applied in a mouse model with a mouse-adapted SARS-CoV-2 strain, which differs in disease course and pathogenesis from human isolates. Transfer of a monoclonal IgM (rather than the polyclonal preparation used here) likely also facilitated delivery of a highly neutralizing, SARS-CoV-2-Spike specific antibody to the site of challenge.

An alternative hypothesis for the lack of IgM-mediated protection is differential rates of antibody clearance. The half-life of infused IgM is typically less than that of IgG, likely due to the fact that IgM does not interact with the recycling Fc receptor (FcRn) (38). The similar concentrations of IgM and IgG present in serum at the time of challenge suggests, however, that the differential persistence of the two antibody isotypes in circulation may not have been a factor in the difference in protection conferred. Alternatively, the IgM’s lack of effectiveness may suggest that another function besides neutralization is responsible for the protection. While IgM mediates complement activation better than IgG it does not interact with Fcγ receptors, therefore effector functions such as antibody dependent cellular cytotoxicity are likely limited (38), although the functions of IgM are not well understood and further research is needed into this area. Such Fc effector functions may be contributing to the IgG-mediated protection observed here, as some reports have linked Fc receptor functions with the protective efficacy of antibodies (65–72).

This study adds to the growing body of literature supporting the importance of neutralizing antibodies for protection against SARS-CoV-2, and confirms that polyclonal sera can be an effective preventive strategy if the neutralizing titers are sufficiently high. As the number of individuals vaccinated against or infected with SARS-CoV-2 grows, the ability to source sera with these neutralizing titers is likely to increase. As new variants emerge that escape vaccine-elicited immunity or monoclonal antibody efficacy, the use of polyclonal immunoglobulin preparations from individuals who have recovered from infection with a new virus variant may be an efficacious tool in the absence of other prevention or treatment strategies. While IgM of high neutralizing titer should in principle be effective, it was unable to protect *in vivo*, suggesting that antibody trafficking or some other function of IgM is more limited than that of IgG and that factors other than neutralization titers should be further investigated as contributing to protection against infection and disease.

## Data availability statement

The raw data supporting the conclusions of this article will be made available by the authors, without undue reservation.

## Ethics statement

The studies involving human participants were reviewed and approved by Walter Reed Army Institute of Research institutional review board. The patients/participants provided their written informed consent to participate in this study. The animal study was reviewed and approved by Animal Care and Use Committee of the US Army Medical Research and Development Command (USAMRDC, proposal number DM170728) Animal Care and Use Review Office and the Institutional Animal Care and Use Committee of Bioqual, Inc. (protocol number 20-079).

## Author contributions

HK, VD, SJK, DLB and KM designed the study. VD, LM-R, BMS, UT, NJ, MZ, ET-F, CHK, SS, AA, CK and DB performed immunologic and virologic assays. MP, BS and SK led the clinical care of the animals. EB performed histopathology. HK, VD, EB, LW, GG, VP, SJK, DLB and KM analyzed and interpreted the data. HK wrote the paper with assistance from all coauthors. All authors contributed to the article and approved the submitted version.
