# Supplementary material for: Convalescent human IgG, but not IgM, from COVID-19 survivors confers dose-dependent protection against SARS-CoV-2 replication and disease in hamsters
Source: Front Immunol. 2023 Mar 21;14:1138629. doi: 10.3389/fimmu.2023.1138629 (PMC10070741; doi:10.3389/fimmu.2023.1138629)
Supplement: Supplementary file 1 [file DataSheet_1.pdf]

Table S1 – Sequences of primers and probes used for quantification of total and subgenomic messenger RNA

Primer/probe name	Sequence (5’-3’)
2019-nCoV_N1-F	GACCCCAAAATCAGCGAAAT
2019-nCoV_N1-R	TCTGGTTACTGCCAGTTGAATCTG
2019-nCoV_N1-P	FAM-ACCCCGCATTACGTTTGGTGGACC-BHQ1
SG-F	CGATCTTGTAGATCTGTTCCTCAAACGAA
SG-R	ATATTGCAGCAGTACGCACACACA
Probe	FAM-ACACTAGCCATCCTTACTGCGCTTCG-BHQ

A

Donor #	Sex	Age group	Symptoms	Days post PCR+	ID50 titer	ID80 titer
1	M	50-60	headache, body ache, fatigue	41	< 40	< 40
2	M	50-60	sharp sore throat, decrease sense of smell	51	149	< 40
3	M	50-60	fever, chills, body ache	53	5,850	461
4	M	50-60	fever, chills	52	761	163
5	M	70-80	asymptomatic	39	1,594	114
6	F	30-40	fever, cough, shortness of breath, flu-like	38	< 40	< 40
7	F	50-60	fever, cough, loss of smell and taste	27	2,647	489
8	M	30-40	unknown	17	< 40	< 40
9	F	30-40	fever, cough, headache	45	851	128
10	M	60-70	asymptomatic	53	2,913	695
11	F	50-60	severe fever, cough	59	206	< 40

B

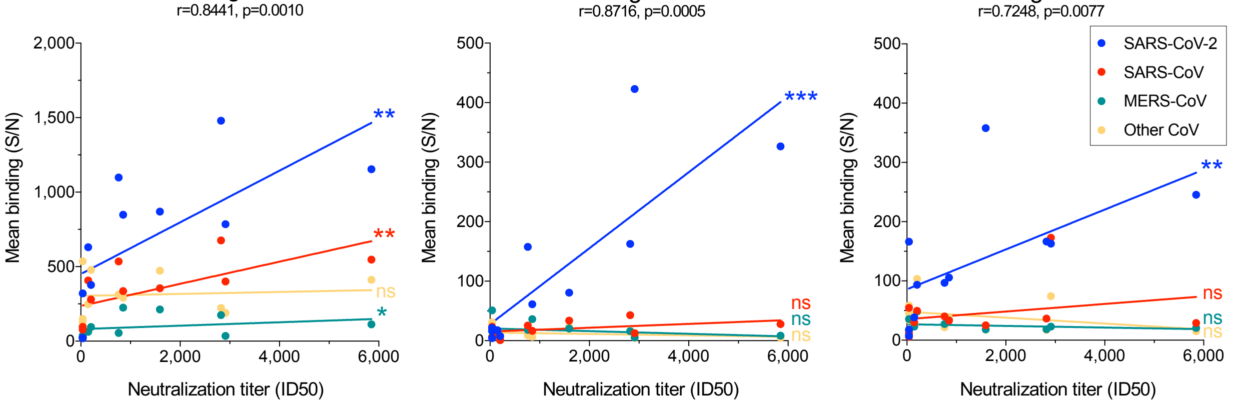

C

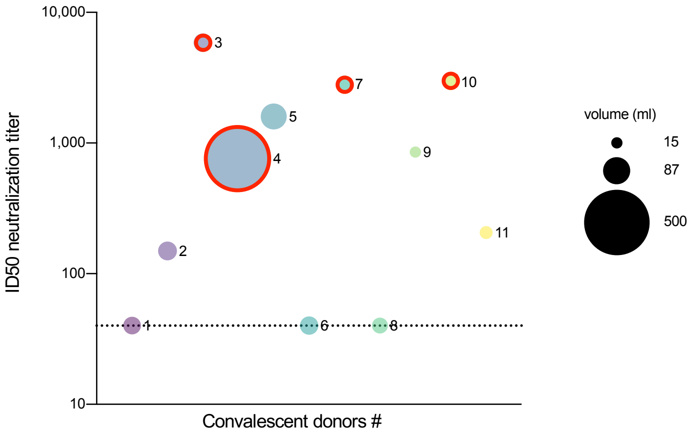

D

	IgG	IgM	IgA
Donor #	IC50 (nM)	IC50 (nM)	IC50 (nM)
3	81.0	1.3	150.9
4	74.7	5.7	302.3
7	49.7	4.9	94.3
10	45.3	2.0	336.5
Geomean	60.8	2.9	195.05

Fig. S1: Binding and neutralizing activities in plasma from convalescent donors. (A) Donor information and plasma neutralization titers at collection time. Time of collection is reported as days post first positive PCR test to SARS-CoV-2. Neutralization was measured in a lentivirus S-pseudotyped assay, both 50% (ID50) and 80% (ID80) inhibitory titers, or reciprocal dilution, are reported. Heat-map shows high (red) to low (green) neutralization activity. Absence of neutralization is indicated in white. (B) Correlation between plasma ID50 neutralization titers and mean binding values to a set of Spike antigens from the indicated CoV for IgG, IgM and IgA. Binding activities, expressed as signal to noise (S/N) relative to a serum control, of each Ig isotype was measured from plasma using a bead-based multiplex assay. For visualization, lines from simple linear regression analysis are shown. Correlation coefficient (Spearman r) and P values are indicated, for SARS-CoV-2 only, below the graph title. Significance of correlation for all antigens are indicated by asterisks (***:p<0.001, **:p<0.01, *:p<0.05, ns, not significant). (C) Donor selection based on plasma neutralization activity and volume of plasma available. Selected donors 3, 4, 7 and 10 are highlighted in circles with red border. (D) Neutralization activity of each Ig isotype for the 4 selected donors. Small-scale purification of IgG, IgM and IgA were performed from 1 ml of plasma and purified isotype were evaluated for neutralization activity. The 50% inhibitory concentration (IC50) is reported in nM for each species.

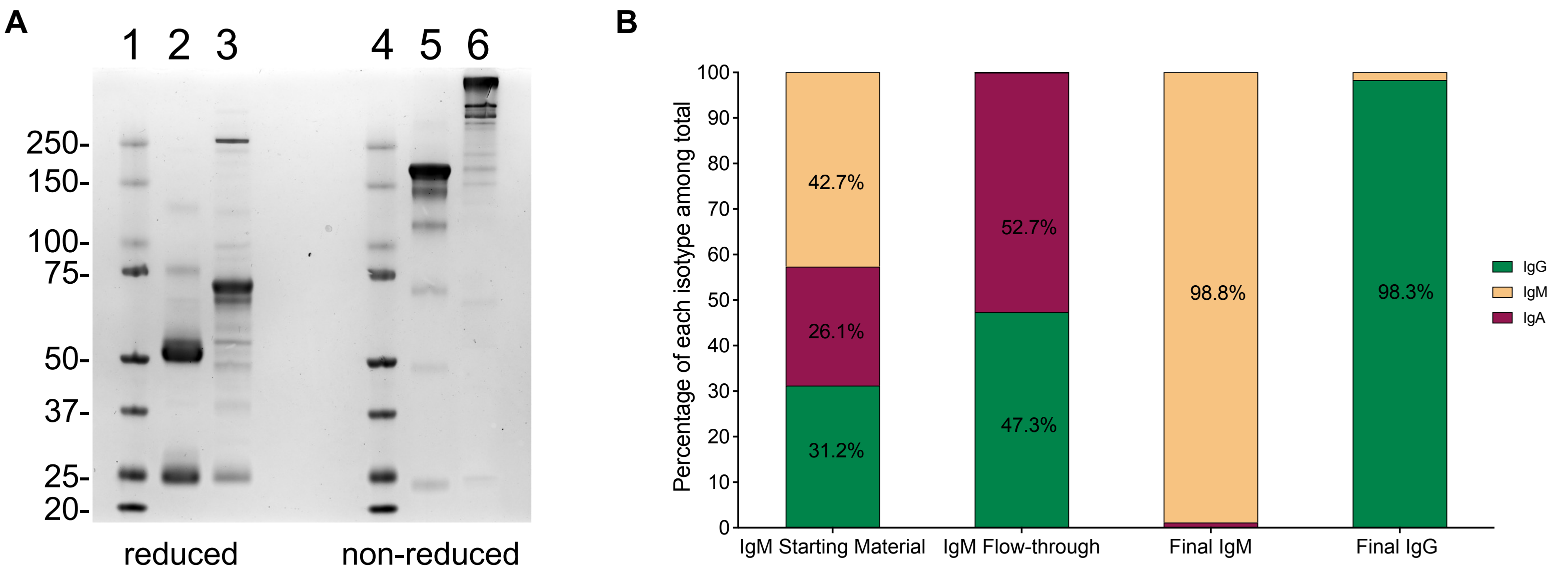

Fig. S2: Assessment of purity and quality of purified IgG and IgM from SARS-CoV-2 convalescent donors. (A) Coomassie staining of purified IgG (lanes 2, 5) and IgM (lanes 3, 6) resolved by SDS-PAGE in reducing (left) and non-reducing (right) conditions. Molecular weights of the protein standard (lanes 1, 4) are indicated in kDa. (B) Purity assessment of convalescent IgG and IgM preparations. Proportion of each Ig isotype in IgM starting material (flow through from IgG purification supplemented with fresh plasma), IgM column flow-through, and final material for IgM and IgG preparations was determined by ELISA.

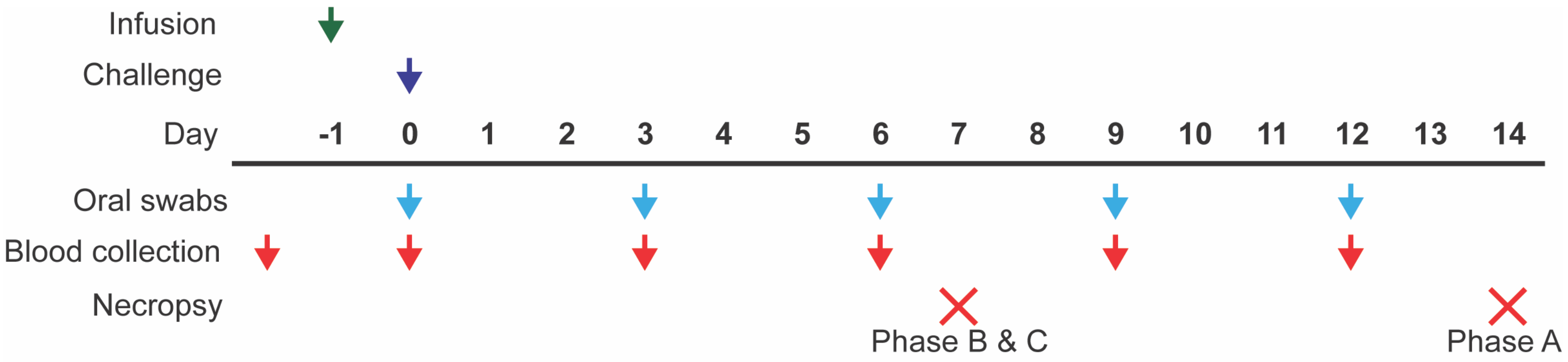

Phase A:

- Convalescent IgG, 100mg/kg
- Naïve IgG, 100mg/kg
- No Ab

Phase B:

- Convalescent IgG, 500mg/kg
- Convalescent IgG, 250mg/kg
- Convalescent IgG, 50mg/kg
- Convalescent IgG, 10mg/kg
- Naïve IgG, 100mg/kg
- No Ab

Phase C:

- Convalescent IgM, 100mg/kg
- Convalescent IgM, 50mg/kg
- No Ab

Figure S3: Infusion and challenge schedule. Animals were infused with either convalescent IgG or IgM at the doses indicated on day -1. Control animals received IgG purified from naïve individuals or no antibody (n=6/group). 24 hours later at day 0 animals were challenged with 1.99×10^4 $TCID_{50}$ SARS-CoV-2 (WA1/2020) via the intranasal route. Oral swabs and blood samples were collected at the times indicated. Animals were necropsied at either day 7 or day 14, and blood and tissue samples collected for pathology and viral load measurements.

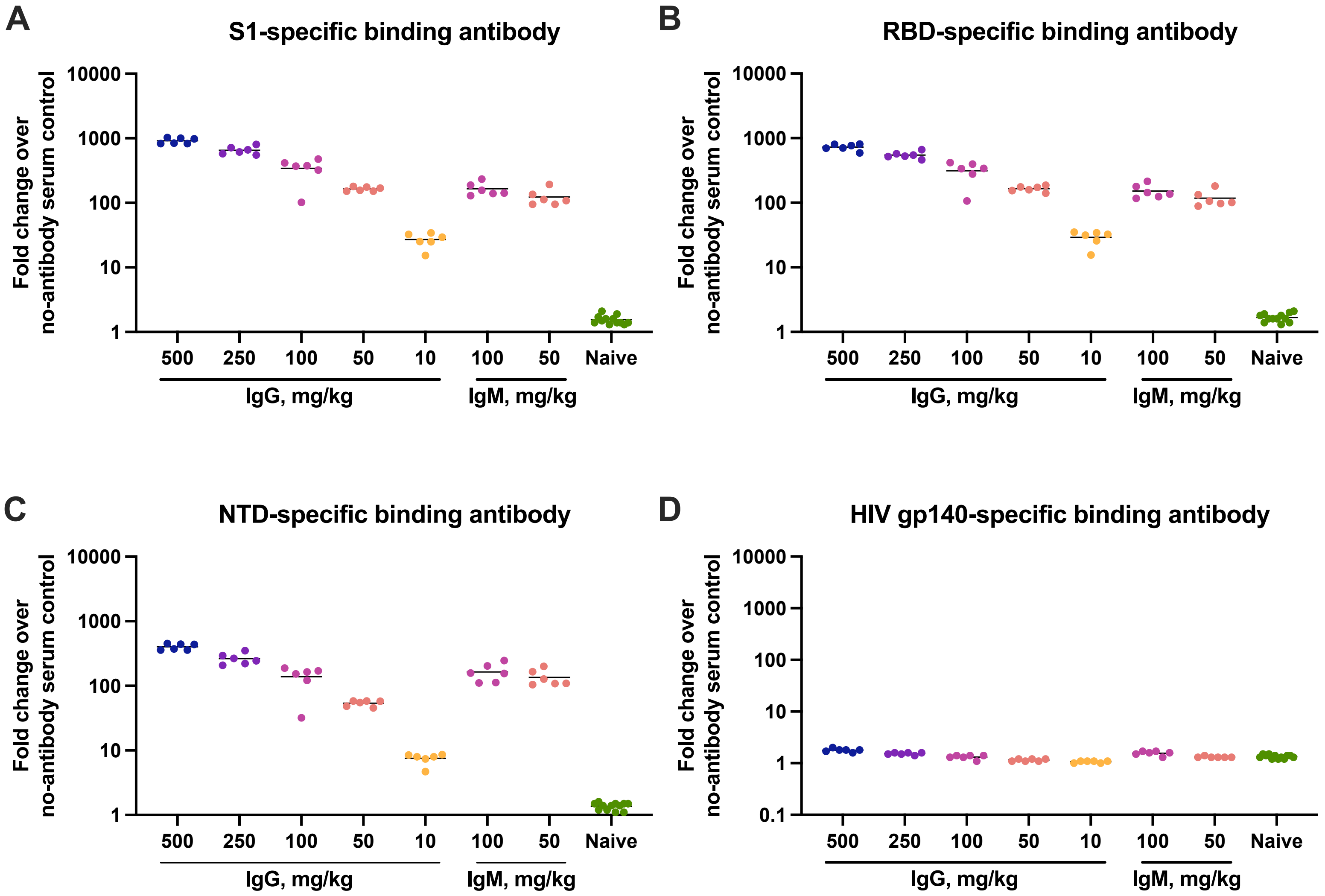

Figure S4: Binding antibody titers at time of challenge in serum to SARS-CoV-2 subunit antigens. Titers of infused antibodies were measured in hamster serum at the time of challenge (study day 0) by multiplex antibody binding assay to the S1 subunit (A), RBD (B), NTD (C) and HIV gp140 (D) antigens. Horizontal bars indicate group means.

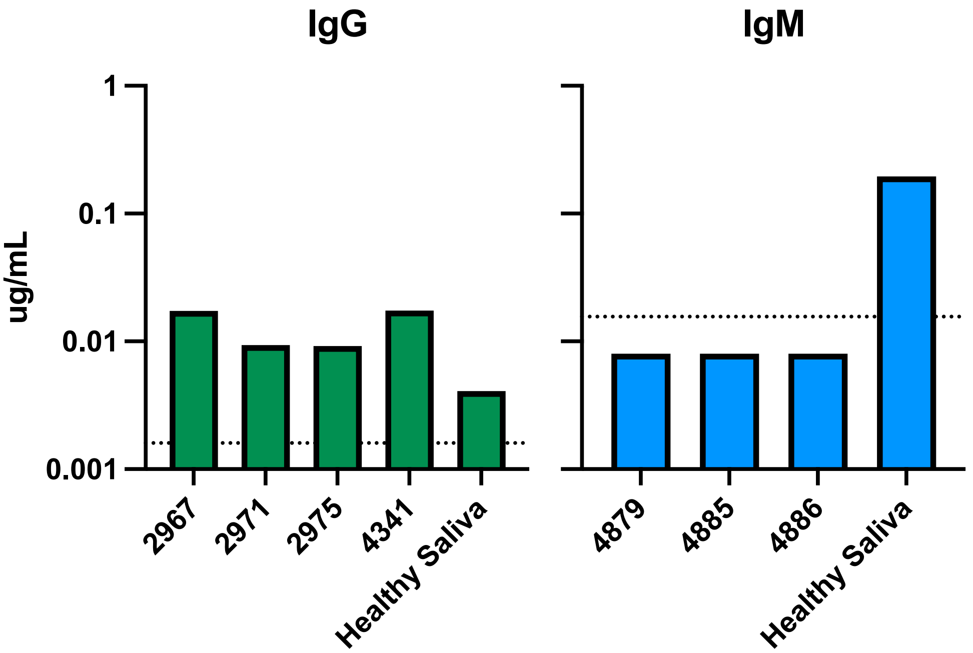

Figure S5: Total antibody titers at time of challenge in oral swabs. Titers of infused antibodies were measured in hamster oral swabs at the time of challenge (study day 0) by human IgG or IgM ELISA assays. Samples were concentrated 3-4 fold prior to measurement. Healthy saliva was a commercially acquired normal pooled human saliva sample. Dotted line indicates the lower limit of detection for each assay.

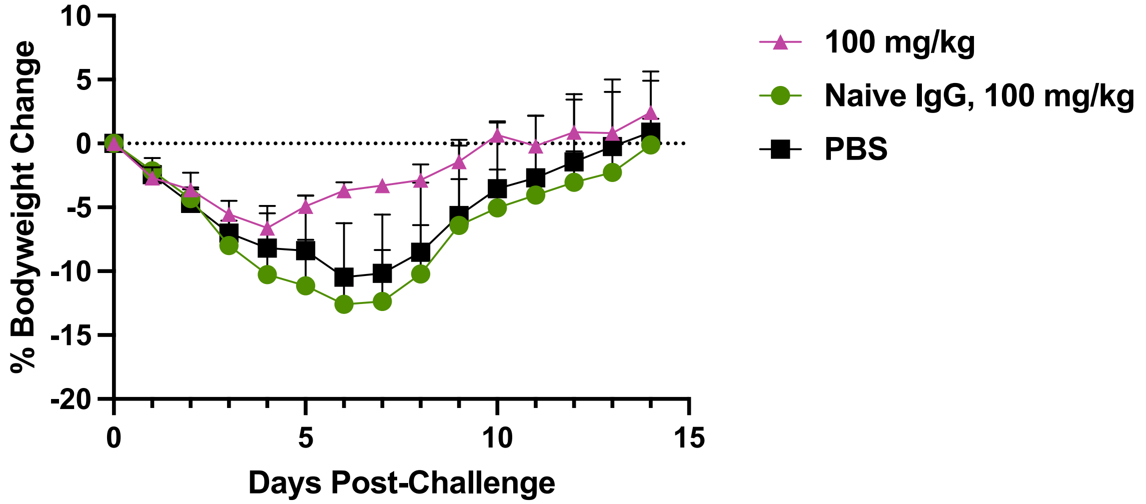

Figure S6: Body weight changes for 14 days following SARS-CoV-2 challenge. Hamster weight was recorded daily in hamsters from the time of viral challenge until necropsy at day 14 (Phase A). Graphs show the mean body weight change for each each group. Error bars indicate standard deviation.

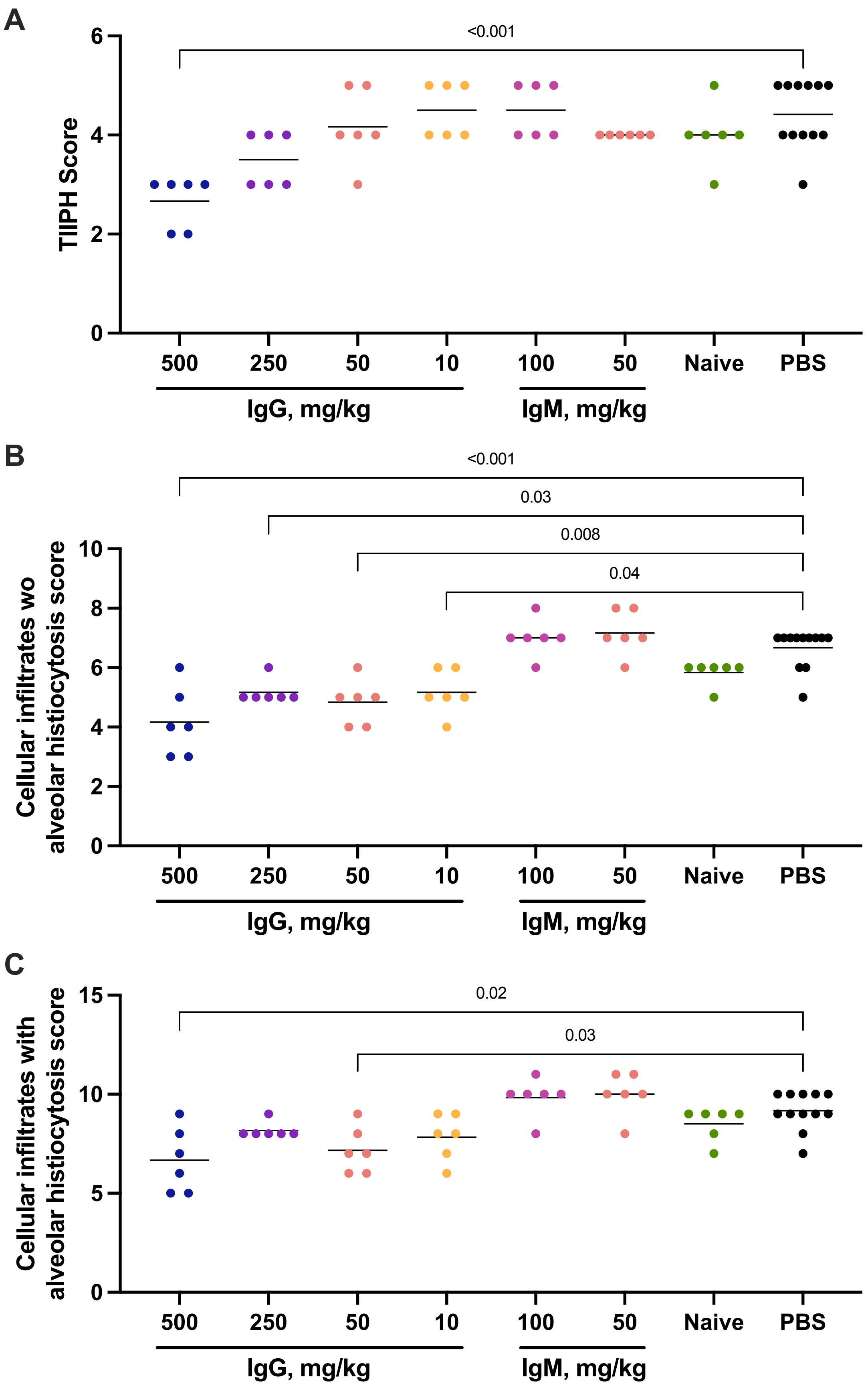

Figure S7: Pathology 7 days post-challenge. Histopathologic examination post-challenge. Lung tissues were collected at necropsy on day 14 post-challenge, fixed with neutral buffered formalin, and stained with hematoxylin and eosin (H&E) for standard microscopic examination. H&E stained slides were scored for pathologic effects in SARS-CoV-2 challenged hamsters. Graphs represent the scoring for type II pneumocyte hyperplasia (A), cellular infiltrates without alveolar histiocytosis (B) and cellular infiltrates including alveolar histiocytosis (C). Dots represent individual animals and the horizontal bar is the mean group score. Each group was compared to the control group that received no antibody infusion by Kruskal-Wallis test with Dunn’s posttest and bars indicate significant differences (P<0.05).

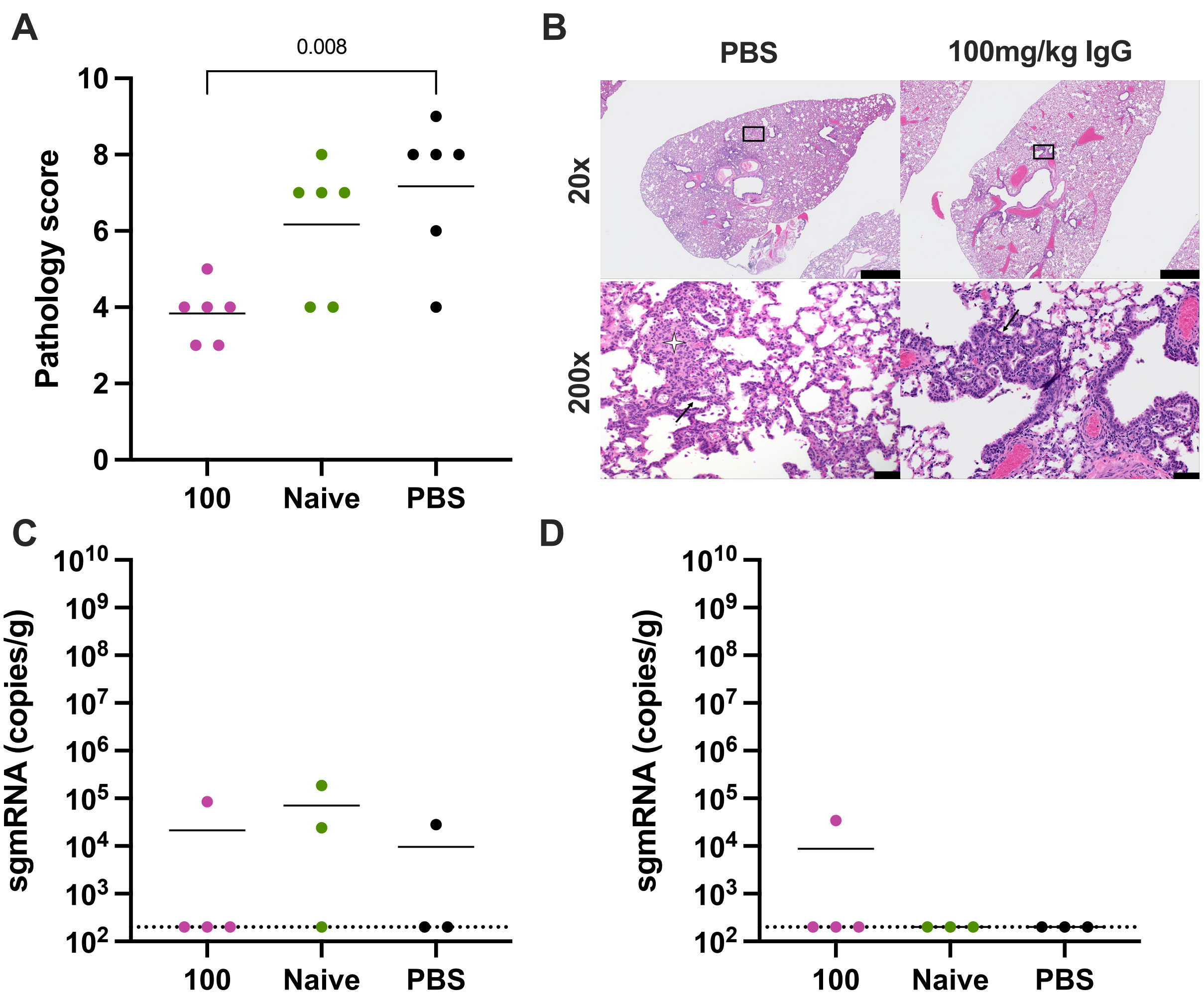

Figure S8: Lung pathology and viral loads 14 days post-challenge. (A) Histopathologic examination post-challenge. Lung tissues were collected at necropsy on day 14 post-challenge, fixed with neutral buffered formalin, and stained with hematoxylin and eosin (H&E) for standard microscopic examination. H&E stained slides were scored for pathologic effects (see Methods) in SARS-CoV-2 challenged hamsters. Dots represent individual animals and the horizontal bar is the mean group score. Significance was assessed by a Kruskal-Wallis test with Dunn's posttest, comparing each group to the control group that received no antibody infusion. (B) Representative lung tissue sections from the 14 day PBS control, and 14 day 100 µg/kg IgG challenged hamsters in the columns as indicated. Rows are given by H&E at 20 and 200 times magnification power (20X and 200X, respectively). The black boxes in the top row indicate the area magnified in the bottom row. Interstitial pneumonia is characterized by inflammatory cellular infiltrates and type II pneumocyte hyperplasia (thick arrow). Scale bars: Top row, 1 mm; bottom row, 50 µm. (C & D) Subgenomic RNA viral loads were measured in lung tissue (C) and nares (D) extracted at necropsy 14 days post-challenge. Lines indicate the group means. Each group was compared to the control group that received no antibody infusion by Kruskal-Wallis test with Dunn's posttest and bars indicate significant differences (P<0.05).

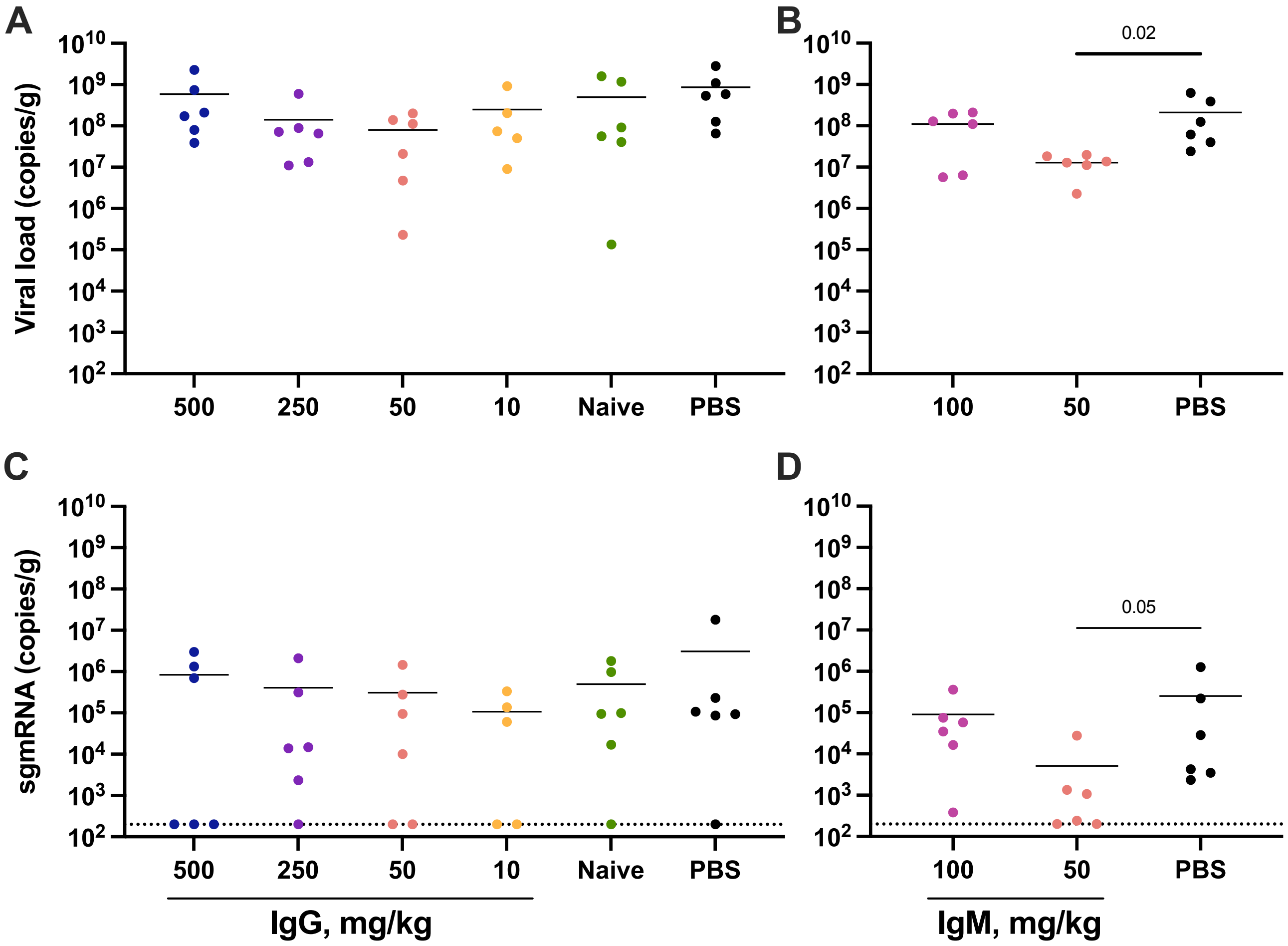

Figure S9: Nares viral loads post-challenge. Nares were collected at necropsy on day 7 post-challenge for viral RNA extraction. Total viral loads were measured by PCR for IgG (A) and IgM (B) animals, and subgenomic RNA measured for IgG (C) and IgM (D) animals. Horizontal bars indicate the group means. Significance was assessed by a Kruskal-Wallis test with Dunn's posttest, comparing each group to the control group that received no antibody infusion.

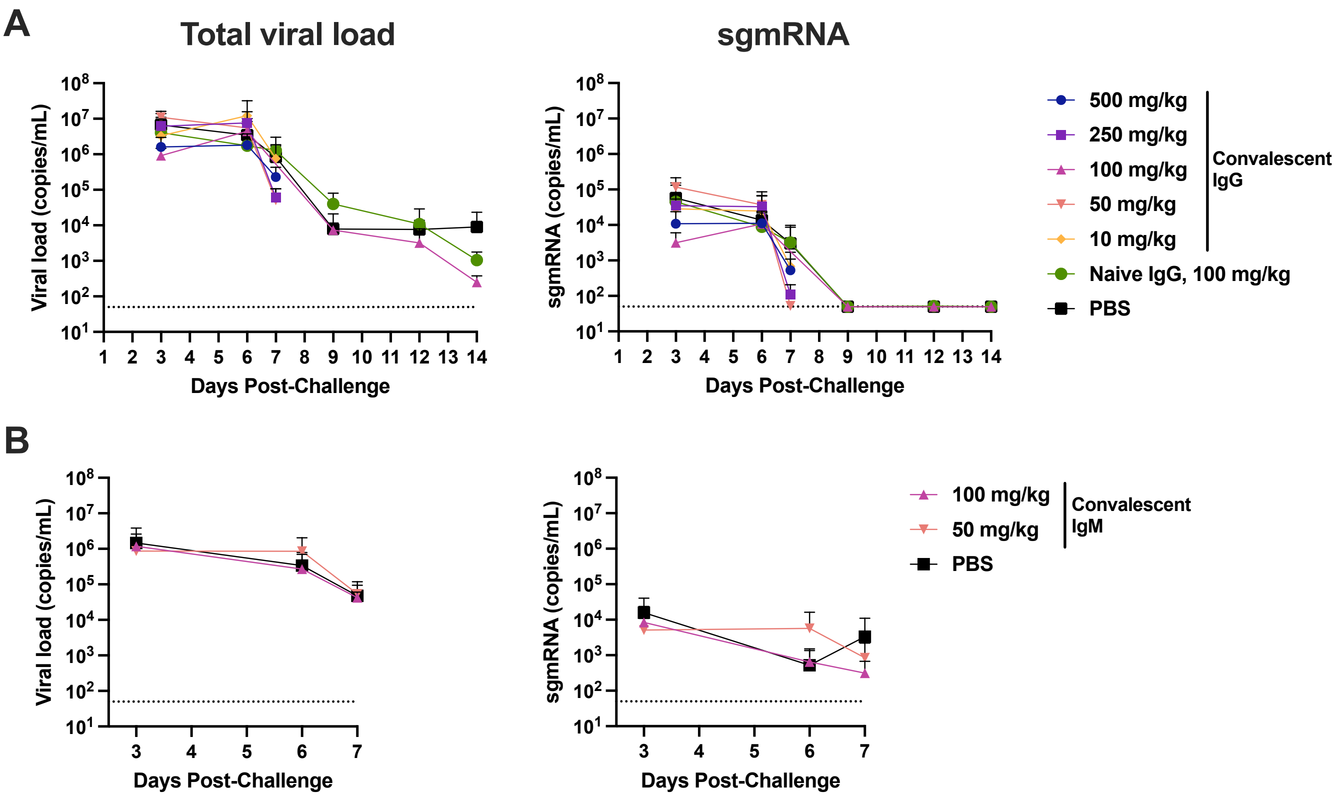

Figure S10: SARS-CoV-2 RNA burden in the oral cavity following challenge. Viral loads were measured in oral swabs collected sequentially following challenge with SARS-CoV-2. Graphs show the mean body viral load for each each group in the animals that received passively transferred IgG (A) or IgM (B). Error bars indicate standard deviation.
